# ZC3HAV1 facilitates STING activation and enhances inflammation

**DOI:** 10.1038/s42003-024-07116-2

**Published:** 2024-10-30

**Authors:** Danhui Qin, Hui Song, Caiwei Wang, Xiaojie Ma, Yu Fu, Chunyuan Zhao, Wei Zhao, Lei Zhang, Weifang Zhang

**Affiliations:** 1 Key Laboratory for Experimental Teratology of the Chinese Ministry of Education, and Key Laboratory of Infection and Immunity of Shandong Province, School of Basic Medical Science, Qilu Hospital, Cheeloo College of Medicine, Shandong University, Jinan, Shandong China; 2 Department of Rheumatology and immunology, Affiliated Hospital of Shandong University of Traditional Chinese Medicine, Jinan, Shandong China; 3 Department of Orthopedic Surgery, the First Affiliated Hospital of Shandong First Medical University & Shandong Provincial Qianfoshan Hospital, Shandong Key Laboratory of Rheumatic Disease and Translational Medicine, Jinan, Shandong China

**Keywords:** Infection, Toll-like receptors

## Abstract

Stimulator of interferon genes (STING) is vital in the cytosolic DNA-sensing process and critical for initiating the innate immune response, which has important functions in host defense and contributes to the pathogenesis of inflammatory diseases. Zinc finger CCCH-type antiviral protein 1 (ZC3HAV1) specifically binds the CpG dinucleotides in the viral RNAs of multiple viruses and promotes their degradation. ZAPS (ZC3HAV1 short isoform) is a potent stimulator of retinoid acid-inducible gene I (RIG-I) signaling during the antiviral response. However, how ZC3HAV1 controls STING signaling is unclear. Here, we show that ZC3HAV1 specifically potentiates STING activation by associating with STING to promote its oligomerization and translocation from the endoplasmic reticulum (ER) to the Golgi, which facilitates activation of IRF3 and NF-κB pathway. Accordingly, *Zc3hav1* deficiency protects mice against herpes simplex virus-1 (HSV-1) infection- or 5,6-dimethylxanthenone-4-acetic acid (DMXAA)-induced inflammation in a STING-dependent manner. These results indicate that ZC3HAV1 is a key regulator of STING signaling, which suggests its possible use as a therapeutic target for STING-dependent inflammation.

## Abstract (web summary)

ZC3HAV1 specifically enhances STING signaling responses to activate IRF3 and NF-κB pathway provides a potential ZC3HAV1-STING-targeting strategy for the clinical management of of STING-dependent inflammatory diseases.

## Introduction

Stimulator of interferon genes (STING) is a crucial component of innate immunity that senses the second messenger cyclic GMP-AMP (cGAMP) and initiates the host defense against pathogens. The cytosolic DNA receptor cGAMP synthase (cGAS) recognizes pathogen-derived DNA or self-DNA from genomic DNA damage and then produces cGAMP to activate STING (also known as TMEM173, MITA, ERIS and MPYS)^1–7^. Following binding of cGAMP, STING oligomerizes and translocates from the endoplasmic reticulum (ER) to the Golgi apparatus, where it recruits and activates TANK-binding kinase 1 (TBK1), which phosphorylates the transcription factor interferon regulatory factor 3 (IRF3), leading to the dimerization and translocation of IRF3 to the nucleus to induce the expression of type I interferons (IFNs-I including IFN-α and IFN-β)^8,9^. The binding of IFNs-I to receptors on the cell surface initiates the Janus kinase/signal transducer and activator of transcription (JAK/STAT) pathway to induce the expression of a variety of interferon-stimulated genes (ISGs) and exert a potent antiviral state^10^. Another signaling module used by STING is the NF-κB-associated pathway. NF-κB usually resides in the cytoplasm of resting cells through association with inhibitors of κB (IκB). STING activates the IκB kinase (IKK) complex, which consists of the catalytic subunits IKKα and IKKβ as well as the regulatory subunit NEMO, and phosphorylates IκB and targets it for degradation, leading to the release of NF-κB from the inhibitory complex and the translocation of NF-κB subunits P50 and P65 to the nucleus, where they act as a transcription factor to induce the expression of proinflammatory cytokines, such as tumor necrosis factor-alpha (TNF-α) and interleukin-6 (IL-6)^11–13^. Therefore, STING plays vital roles in antiviral and inflammatory responses, and its activity should be tightly controlled to potentiate the host defense against pathogens and avoid detrimental effects.

STING forms a dimer on the ER membrane, and the dimerization of STING is essential for TBK1 recruitment and subsequent IFN-β induction^5^. Mounting evidence reveals an important role of STING dimerization in the innate immune response. Therefore, many molecules positively regulate STING dimerization to activate downstream signaling pathways. For example, the E3 ubiquitin ligase tripartite motif 56 (TRIM56) preferentially catalyzes the K63-linked ubiquitination of STING after stimulation with dsDNA, which induces STING dimerization to potentiate the antiviral response^14^. Transmembrane emp24 protein transport domain containing 2 (TMED2) and TIR-domain containing adaptor-inducing IFN-β (TRIF) reinforce STING dimerization and facilitate its trafficking by directly interacting with STING against herpes simplex virus 1 (HSV-1) infection^15,16^. Moreover, some viruses have developed strategies to inhibit STING dimerization to avoid host immune activation. The HCMV tegument protein UL94 associates with STING and suppresses its dimerization to evade the immune response and establish latent infection^17^.

Zinc finger CCCH-type antiviral protein 1 (ZC3HAV1), also known as zinc finger antiviral protein (ZAP), is a cell-intrinsic antiviral factor induced by both IFNs-I and viruses^18–20^. ZC3HAV1 restricts the replication of multiple viruses, including alphaviruses, filoviruses and influenza A virus (IAV)^21–23^. ZC3HAV1 also binds to the genome of human immunodeficiency virus (HIV) to attenuate HIV replication^24^. Numerous studies have revealed that the zinc fingers of ZC3HAV1 specifically bind CpG dinucleotides in viral RNAs to promote their degradation or translation inhibition^25–30^. On the basis of RNA features such as the number of CpG, juxtaposition, and surrounding sequence that ZC3HAV1 specifically recognizes, mutations in RNA viruses, which have an increased sensitivity to ZC3HAV1, can be designed to generate attenuated viral vaccines^31^. ZC3HAV1 possesses two alternatively spliced isoforms that differ in their C-terminus, referred to as ZAPL (the long isoform) and ZAPS (the short isoform)^22^. Both ZAPL and ZAPS share an identical N-terminal four CCCH-type zinc finger RNA-binding domain (RBD)^32^. ZAPL contains a catalytically inactive C-terminal poly (ADP ribose) polymerase (PARP)-like domain, and this PARP domain makes ZAPL a more potent viral inhibitor compared to ZAPS^33^. ZAPS is selectively induced by 5’-triphosphate-modified RNA (3pRNA) and acts as a positive regulator of the retinoic acid-inducible gene I (RIG-I)-like receptors (RLRs)-dependent RNA sensing pathway by promoting the oligomerization of RIG-I^34^. However, the potential role of ZC3HAV1 in STING-dependent inflammation and the DNA-sensing pathway remains unknown.

Here we showed that *Zc3hav1* deficiency impaired the innate immune response induced by HSV-1 (a type of DNA virus that activates cGAS), ISD (interferon-stimulatory DNA recognized by cGAS), cGAMP (endogenous STING ligand), or DMXAA (5,6-dimethylxanthenone-4-acetic acid, STING agonist). Concordantly, *Zc3hav1* deficiency attenuated the antiviral response against HSV-1 and STING-dependent inflammation in vivo. Mechanistically, ZC3HAV1 interacted with STING and facilitated the oligomerization and trafficking of STING from the ER to the Golgi. These results indicated that ZC3HAV1 is critical for STING activation and suggest that it is a priming target for the treatment of diseases caused by aberrant STING activity.

## Results

### ZC3HAV1 enhances the cGAS-STING signaling

**Fig. 1:**
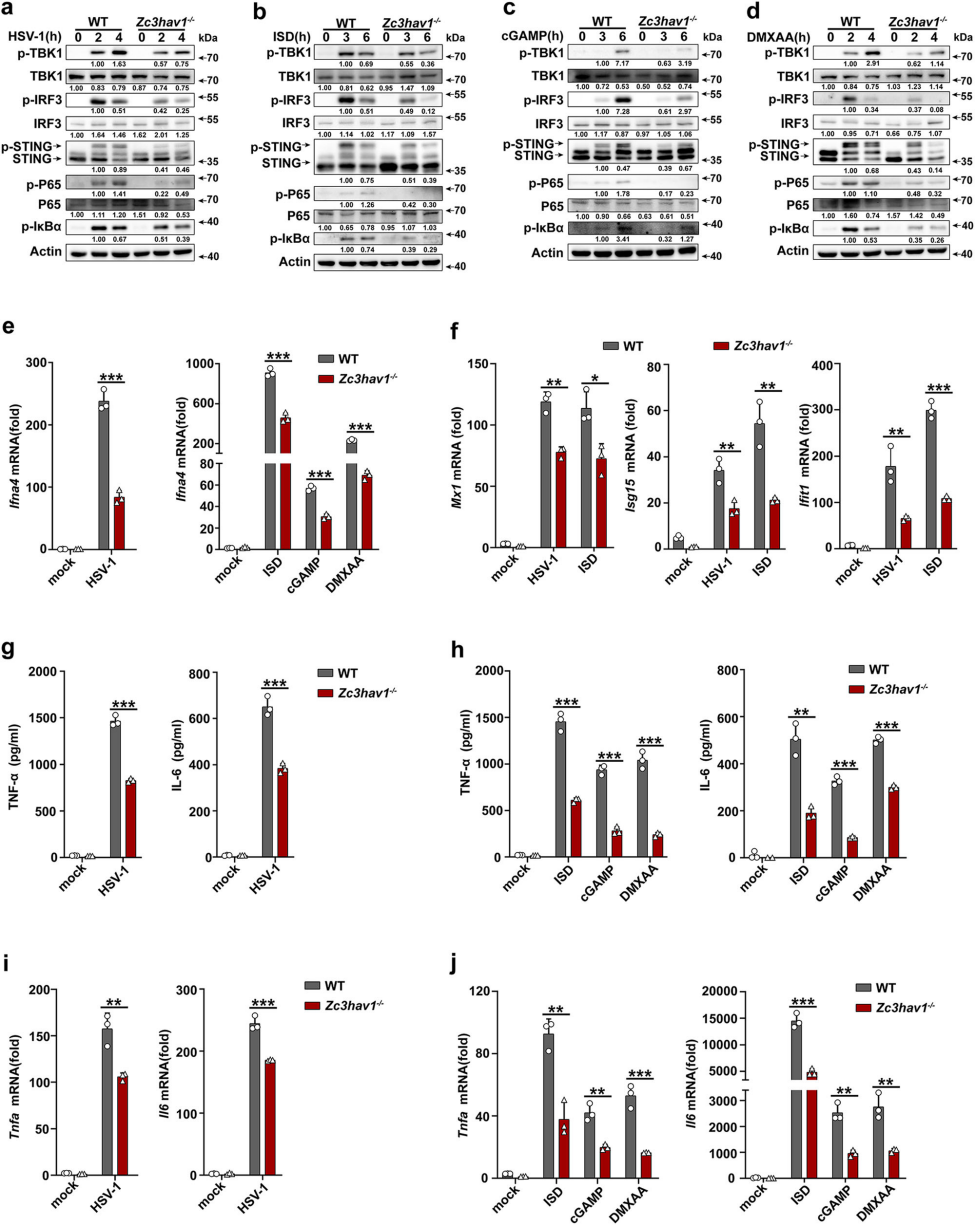
*Zc3hav1* deficiency inhibits the cGAS-STING pathway. **a**–**d** Immunoblot analysis of phosphorylated (p-) and total TBK1, IRF3, STING, P65 and IκBα protein in PMs from WT and *Zc3hav1*^-/-^ mice infected with HSV-1 or stimulated with ISD, cGAMP or DMXAA. **e** RT-PCR analysis of *Ifna4* mRNA levels in PMs from WT and *Zc3hav1*^-/-^ mice infected with HSV-1 or stimulated with ISD, cGAMP or DMXAA. **f** RT-PCR analysis of *Mx1, Isg15* and *Ifit1* mRNA levels in PMs from WT and *Zc3hav1*^-/-^ mice infected with HSV-1 or stimulated with ISD. **g**, **h** ELISA analysis of TNF-α and IL-6 secreted from PMs of WT and *Zc3hav1*^-/-^ mice infected with HSV-1 or stimulated with ISD, cGAMP or DMXAA. **i**, **j** RT-PCR analysis of *Tnfa and Il6* mRNA levels in PMs from WT and *Zc3hav1*^-/-^ mice infected with HSV-1 or stimulated with ISD, cGAMP or DMXAA. All data are represented as means ± SD. All experiments were repeated at a minimum of three times. Statistical significance was determined by unpaired two-tailed Student’s *t* test: *P ≤ 0.05, **P ≤ 0.01, and *** *P* ≤ 0.001.

We first characterized the potential function of ZC3HAV1 in the cGAS-STING pathway. *Zc3hav1*^*-/-*^ mice were generated and confirmed (Supplementary Fig. 1a). The phosphorylation of STING and downstream signaling molecules, TBK1 and IRF3 was increased upon HSV-1 infection, or ISD, cGAMP, or DMXAA stimulation in mouse peritoneal macrophages (PMs), whereas *Zc3hav1* deficiency attenuated the phosphorylation of STING, TBK1, and IRF3 (Fig. 1a–d). In addition, *Zc3hav1* deficiency reduced HSV-1-, ISD-, cGAMP- or DMXAA-induced phosphorylation of P65 and IκBα. Correspondingly, *Zc3hav1* deficiency suppressed *Ifna4* mRNA expression induced by stimulation with HSV-1, ISD, cGAMP or DMXAA (Fig. 1e). Consistently, the mRNA levels of ISGs such as *Mx1, Isg15 and Ifit1* were attenuated by *Zc3hav1* deficiency after stimulation with HSV-1 or ISD (Fig. 1f). The secretion of TNF-α and IL-6 proteins and the mRNA expression of *Tnfa* and *Il6* were markedly inhibited in *Zc3hav1*^*-/-*^ mouse PMs following HSV-1, ISD, cGAMP or DMXAA stimulation (Fig. 1g–j).

To further confirm the regulatory role of ZC3HAV1 in the cGAS-STING signaling, small interfering RNA (siRNA) knockdown experiments were performed. The mixture of two siRNAs targeting mouse *Zc3hav1*, si*Zc3hav1*-1 and si*Zc3hav1*-2 (designated in the subsequent experiments as si*Zc3hav1*), suppressed endogenous ZC3HAV1 expression in mouse PMs (Supplementary Fig. 1b). The knockdown efficiency of each siRNA and the si*Zc3hav1* mixture was confirmed by assessing *Zc3hav1* mRNA expression (Supplementary Fig. 1c). ZC3HAV1 knockdown considerably inhibited STING-dependent phosphorylation of IRF3, P65 and IκBα in mouse PMs after stimulation with HSV-1, ISD, cGAMP or DMXAA (Supplementary Fig. 1d–i). We reconstituted ZAPS or ZAPL into *Zc3hav1*^*-/-*^ mouse embryonic fibroblasts (MEFs), and showed that both ZAPS and ZAPL restored the phosphorylation of IRF3 after stimulation with ISD or DMXAA (Supplementary Fig. 1j, k), which suggests that both ZAPS and ZAPL may play roles in the STING pathway. The induction of *Ifna4* and *Il6* mRNA in response to stimulation with ISD, cGAMP or DMXAA was markedly impaired by ZC3HAV1 knockdown (Supplementary Fig. 1l, m). The titer of HSV-1 in the PMs of *Zc3hav1*^*-/-*^ mice was then examined by a plaque assay. *Zc3hav1* deficiency enhanced HSV-1 replication (Supplementary Fig. 1n). Collectively, these data indicated that ZC3HAV1 may be important for the STING-mediated signaling pathway by enhancing the activation of both the IRF3 pathway and the NF-κB pathway.

### ZC3HAV1 has no effect on TLR activation

**Fig. 2:**
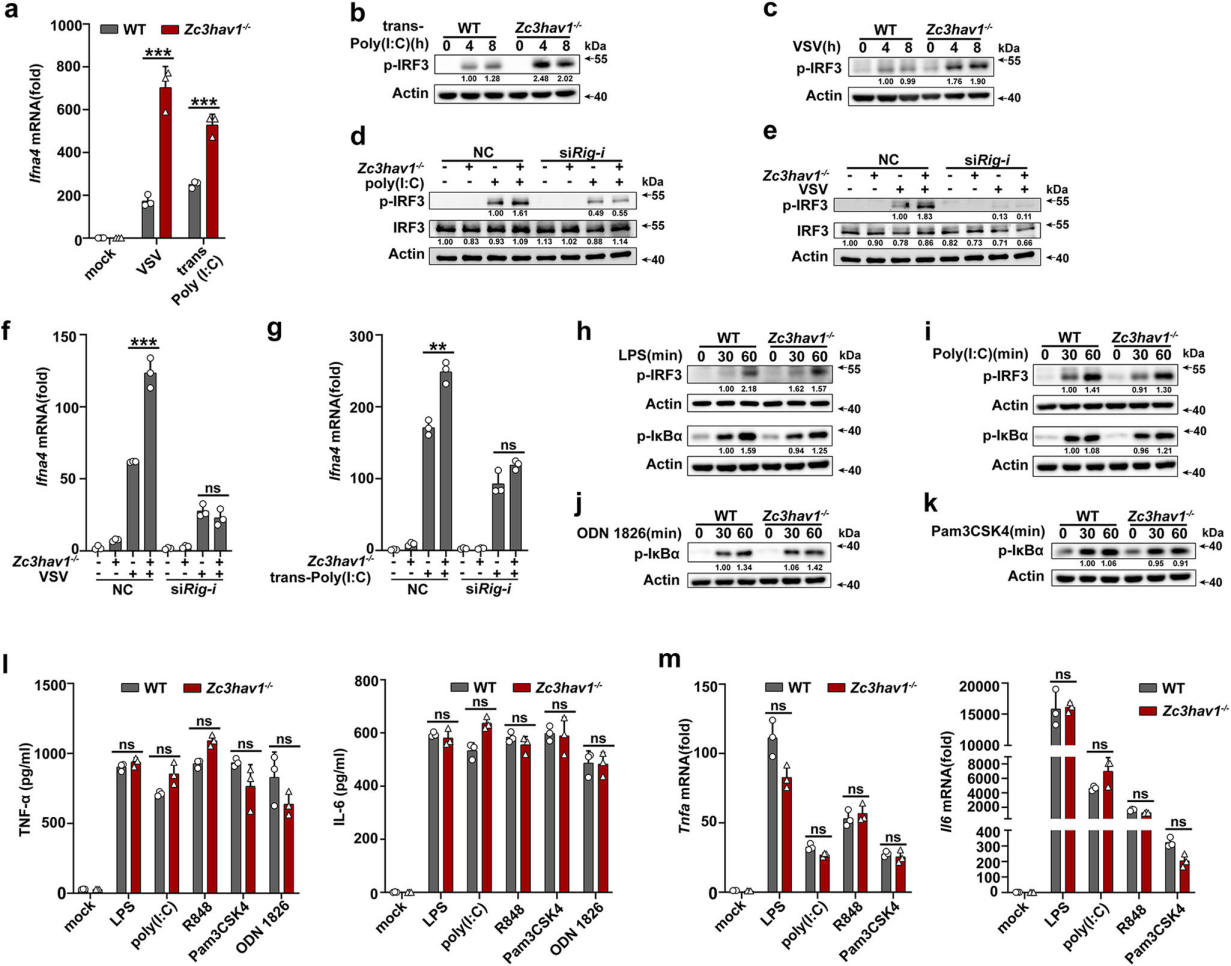
ZC3HAV1 has no effect on TLR activation. **a** RT-PCR analysis of *Ifna4* mRNA levels in VSV-stimulated or poly(I:C)-transfected PMs from WT and *Zc3hav1*^-/-^ mice. **b**, **c** Immunoblot analysis of IRF3 phosphorylation in PMs from WT and *Zc3hav1*^-/-^ mice stimulated with VSV or transfected with poly(I:C). **d**, **e** Immunoblot analysis of p-IRF3 and total IRF3 protein in PMs from WT and *Zc3hav1*^-/-^mice transfected with control (NC) siRNA or *Rig-i* siRNA for 48 h and subsequently stimulated with VSV or transfected with poly(I:C). **f**, **g** RT-PCR analysis of *Ifna4* mRNA levels in PMs from WT and *Zc3hav1*^-/-^ mice transfected with NC siRNA or *Rig-i* siRNA for 48 h and subsequently stimulated with VSV or transfected with poly(I:C). **h**, **i** Immunoblot analysis of IRF3 and IκBα phosphorylation in PMs from WT and *Zc3hav1*^-/-^ mice stimulated with LPS or poly(I:C). **j**, **k** Immunoblot analysis of IκBα phosphorylation in PMs from WT and *Zc3hav1*^-/-^ mice stimulated with ODN 1826 or Pam3CSK4. **l** ELISA analysis of TNF-α and IL-6 secretion in PMs from WT and *Zc3hav1*^-/-^ mice stimulated with LPS, poly(I:C), R848, Pam3CSK4 or ODN 1826. **m** RT-PCR analysis of *Tnfa* and *Il6* mRNA levels in PMs from WT and *Zc3hav1*^-/-^ mice stimulated with LPS, poly(I:C), R848 or Pam3CSK4. All data are represented as means ± SD. Similar results were obtained from three independent experiments. Statistical significance was determined by an unpaired two-tailed Student’s *t* test: **P ≤ 0.01, *** *P* ≤ 0.001, ns, not significant (*P*  >  0.05).

*Zc3hav1* deficiency enhanced vesicular stomatitis virus (VSV, a ssRNA virus recognized by RIG-I) infection- or the dsRNA analog poly(I:C) transfection-induced *Ifna4* mRNA expression and IRF3 phosphorylation (Fig. 2a–c). We further knocked down RIG-I with siRNA in *Zc3hav1*-deficient cells and stimulated these cells with VSV or poly(I:C) transfection. RIG-I knockdown eliminated the increase in IRF3 phosphorylation and *Ifna4* mRNA expression caused by *Zc3hav1* deficiency (Fig. 2d–g). These results differ from those of another report that showed that ZAPS facilitates RIG-I-mediated signaling^34^. In that study, the researchers focused on the role of ZAPS and conducted in vitro experiments (using human cell lines to overexpress or knock down ZAPS), whereas in the present study, we used *Zc3hav1*^*-/-*^ primary mouse cells in which endogenous ZAPS and ZAPL were both deleted. In another study, a similar result of *Zc3hav1*^*-/-*^ mouse cells presenting increased expression of IFNs-I after stimulation with poly U/UC RNA was observed^35^. Thus, deficiency of both ZAPS and ZAPL enhanced RIG-I-dependent signaling in mouse cells. In contrast, *Zc3hav1* deficiency did not affect the phosphorylation of IRF3 or IκBα after stimulation with LPS (a TLR4 ligand) or poly(I:C) (a TLR3 ligand) (Fig. 2h, i). In addition, *Zc3hav1* deficiency had no influence on ODN 1826 (a TLR9 ligand) -, or Pam3CSK4 (a TLR1/2 ligand)-induced phosphorylation of IκBα (Fig. 2j, k). Furthermore, *Zc3hav1* deficiency did not alter LPS-, poly(I:C)-, R848 (a TLR7/8 ligand)-, Pam3CSK4-, or ODN 1826-induced TNF-α and IL-6 secretion or *Tnfa* and *Il6* mRNA expression (Fig. 2l, m). These data indicated that ZC3HAV1 specifically enhances the cGAS-STING pathway.

### ZC3HAV1 targets STING

**Fig. 3:**
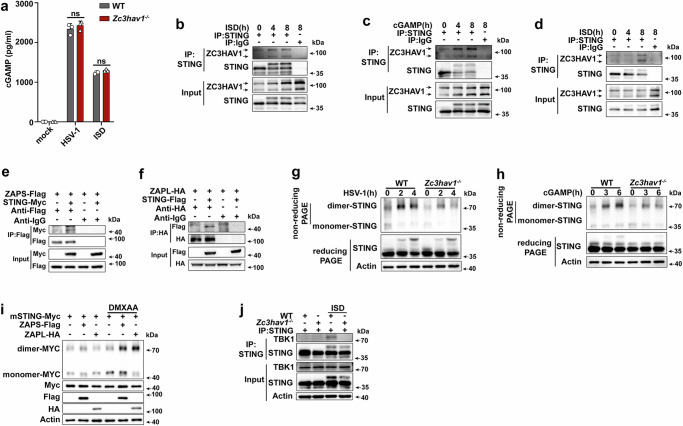
ZC3HAV1 interacts with STING. **a** ELISA analysis of cGAMP production in PMs from WT or *Zc3hav1*^-/-^ mice following HSV-1 infection or ISD stimulation. **b**, **c** Immunoprecipitation (IP) analysis of the endogenous association between ZC3HAV1 and STING in ISD- or cGAMP-stimulated mouse PMs. **d** IP analysis of the endogenous associations between ZC3HAV1 and STING in ISD-stimulated THP-1 cells. **e**, **f** IP analysis of the associations between STING and ZAPS, or ZAPL in HEK293T cells transfected with the indicated plasmids. **g**, **h** Native PAGE and immunoblot analysis of the dimerization of endogenous STING in PMs from WT and *Zc3hav1*^-/-^ mice following HSV-1 infection or cGAMP stimulation. **i** Native PAGE and immunoblot analysis of DMXAA-induced dimerization of STING in HEK293T cells transfected with the indicated plasmids. **j** IP analysis of the recruitment of STING to TBK1 in PMs from WT or *Zc3hav1*^-/-^ mice transfected with ISD. All data are represented as means ± SD. Similar results were obtained from three independent experiments. Statistical significance was determined by an unpaired two-tailed Student’s *t* test: ns, not significant (*P* > 0.05).

As shown above, ZC3HAV1 markedly promoted the STING-mediated signaling pathway activated by the endogenous STING ligand cGAMP and the STING agonist DMXAA. The promoting effect of ZC3HAV1 on STING signaling was not derived by affecting cGAMP levels, since *Zc3hav1* deficiency did not affect the production of cGAMP (Fig. 3a). We then examined whether ZC3HAV1 targets STING to activate the STING-mediated pathway. ZAPS and ZAPL interacted with STING constitutively in its resting state in macrophages and THP-1 cells, and these associations were further enhanced after ISD or cGAMP stimulation (Fig. 3b–d). Consistently, interactions between exogenously expressed Myc-tagged STING and Flag-tagged ZAPS or between Flag-tagged STING and HA-tagged ZAPL were observed in HEK293T cells (Fig. 3e, f). Native PAGE and immunoblot analysis revealed that endogenous STING dimerization in response to stimulation with HSV-1, or cGAMP was decreased in PMs from *Zc3hav1*^-/-^ mice (Fig. 3g, h) or those treated with *Zc3hav1* siRNA and stimulated with HSV-1, ISD, or cGAMP (Supplementary Fig. 2a–c). Consistent with these results, the overexpression of ZAPS or ZAPL in HEK293T cells promoted the dimerization of exogenous STING following DMXAA stimulation (Fig. 3i). The STING C-terminal domain (CTD) binds to its ligands and recruits TBK1, and therefore we assessed the binding of TBK1 to STING in *Zc3hav1-*deficient cells. *Zc3hav1* deficiency abolished the ISD-induced recruitment of TBK1 to STING (Fig. 3j). Taken together, these data corroborated that ZC3HAV1 is associated with STING as part of its involvement in the process of cGAS-STING activation.

### ZC3HAV1 promotes STING oligomerization and trafficking to the Golgi

**Fig. 4:**
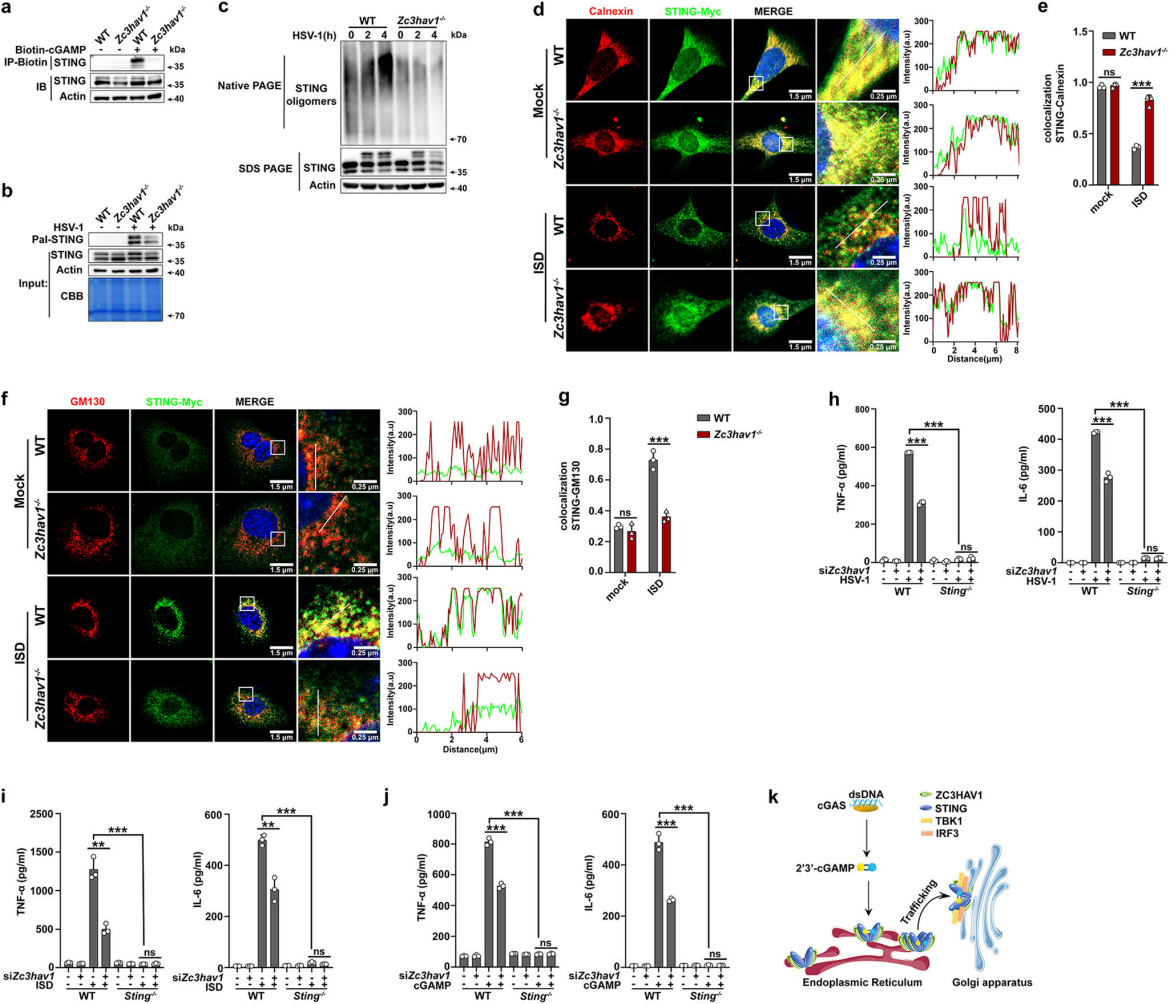
*Zc3hav1* deficiency inhibits STING palmitoylation, oligomerization and ER-to-Golgi trafficking. **a** In vitro pull-down analysis of biotin-cGAMP (C157) binding to STING. **b** Immunoblot analysis of STING palmitoylation by click chemistry in PMs from WT or *Zc3hav1*^-/-^ mice following HSV-1 infection. **c** Native PAGE and immunoblot analysis of STING oligomerization in PMs from WT and *Zc3hav1*^-/-^ mice following HSV-1 infection. **d** Confocal microscopy of MEFs from WT or *Zc3hav1*^-/-^ mice transfected with STING-Myc for 24 h and then stimulated with ISD. The cells were fixed, permeabilized and immunostained for Myc (green), and the ER marker-Calnexin (red). The sections were counterstained with DAPI (blue) to visualize the nuclei. Scale bars, 1.5 and 0.25 μm. The intensity profiles of each line were quantified by ImageJ software. **e** Colocalization analysis of STING and Calnexin by Manders’ Colocalization Coefficients (MCC) analyzed with ImageJ software. **f** Confocal microscopy of MEFs from WT or *Zc3hav1*^-/-^ mice transfected with STING-Myc for 24 h and then stimulated with ISD. The cells were fixed, permeabilized and immunostained for STING (green), and the Golgi marker-GM130 (red). The sections were counterstained with DAPI (blue) to visualize the nuclei. Scale bars, 1.5 and 0.25 μm. The intensity profiles of each line were quantified by ImageJ software. **g** Colocalization analysis of STING and GM130 by MCC analyzed with ImageJ software. **h**–**j** ELISA analysis of TNF-α and IL-6 secreted from PMs of WT and *Sting*^-/-^ mice transfected with NC siRNA or *Zc3hav1* siRNA for 48 h and infected with HSV-1 or stimulated with ISD or cGAMP. **k** Diagram of the role of ZC3HAV1 in the cGAS-STING pathway. All data are represented as means ± SD. One representative image or result from at least three independent experiments was shown. Statistical significance was determined by an unpaired two-tailed Student’s *t* test in (**e**, **g**) and ANOVA test in (**h**–**j**): ** *P* ≤ 0.01, *** *P* ≤ 0.001, ns, not significant (*P* > 0.05).

Binding to cGAMP induces a 180° rotation of STING, which facilitates the oligomerization of STING and its STEEP-mediated translation from the ER to the Golgi^36^. The palmitoylation of STING at Cys88 and Cys91 is essential for STING signaling, and inhibition of STING palmitoylation blocks STING activation^37^. We next determined whether ZC3HAV1 affects these events, which are important for STING signal transduction. As expected, *Zc3hav1* deficiency attenuated the capacity of STING to bind cGAMP (Fig. 4a). *Zc3hav1* deficiency suppressed STING palmitoylation and oligomerization after stimulation with HSV-1 (Fig. 4b, c). To determine the localization of STING further, we stained MEFs with the ER marker Calnexin and the Golgi marker GM130 and examined them via confocal microscopy. *Zc3hav1* deficiency increased the distribution of STING in the ER compartments and correspondingly reduced its distribution in the Golgi compartments after ISD stimulation, which indicates that trafficking of STING from the ER to the Golgi was impaired by *Zc3hav1* deficiency (Fig. 4d–g). Collectively, these results confirmed the vital role of ZC3HAV1 in STING oligomerization and trafficking, which are responsible for the activation of STING and downstream signaling.

To further test whether the impact of ZC3HAV1 on cytokine secretion depends on STING, we transfected *Zc3hav1* siRNA into *Sting*^*-/-*^ mouse PMs. *Sting* deficiency drastically reduced the secretion of TNF-α and IL-6 in response to stimulation with HSV-1, ISD, or cGAMP, and the ZC3HAV1 knockdown-induced inhibition of TNF-α and IL-6 secretion was completely abolished by *Sting* deficiency (Fig. 4h–j). Consistently, the STING inhibitor H-151 markedly impaired TNF-α and IL-6 secretion and counteracted the inhibitory effect on TNF-α and IL-6 secretion caused by *Zc3hav1* deficiency after stimulation with ISD, cGAMP, or HSV-1 (Supplementary Fig. 2d–f). These results that *Zc3hav1* deficiency- or knockdown-mediated inhibition of cytokine secretion was abolished by *Sting* deficiency or STING inhibitor H-151 confirmed that the proinflammatory effect of ZC3HAV1 is STING-dependent. In summary, our results demonstrated that ZC3HAV1 interacts directly with STING to reinforce its oligomerization and facilitate its trafficking from the ER to the Golgi, where it recruits TBK1 and activates IRF3 (Fig. 4k).

### *Zc3hav1* deficiency ameliorates STING-dependent inflammation

**Fig. 5:**
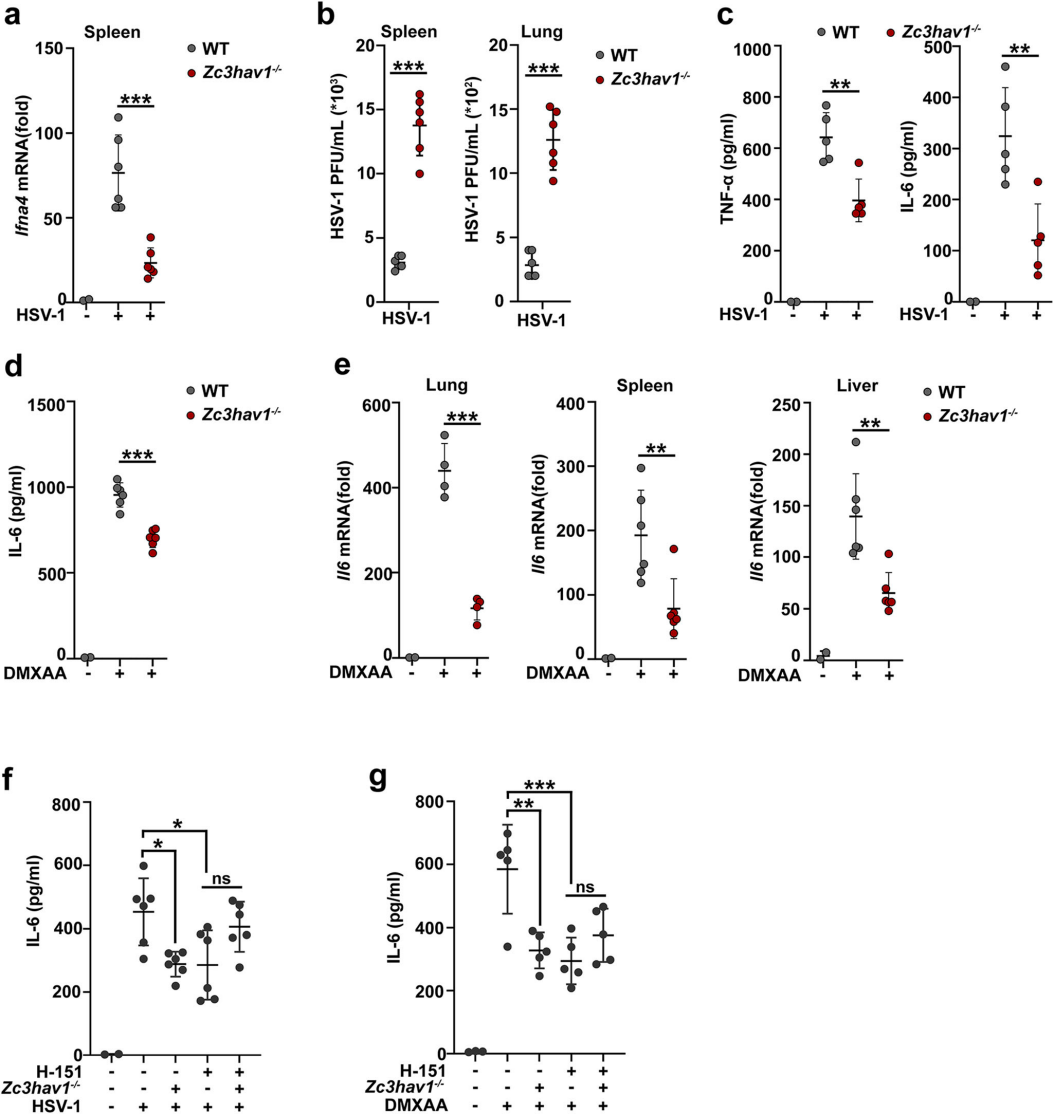
*Zc3hav1* deficiency ameliorates STING-dependent inflammation. **a** RT-PCR analysis of *Ifna4* mRNA levels in spleen tissues from WT or *Zc3hav1*^-/-^ mice 12 h after i.p. injection of HSV-1 (2 × 10^7^ PFU per mouse) (PBS, *n* = 2; HSV-1, *n* = 6 per condition). **b** Plaque assay of the viral titers in spleen and lung tissues from WT or *Zc3hav1*^-/-^ mice 48 h after i.p. injection of HSV-1 (2 × 10^7^ PFU per mouse) (HSV-1, *n* = 6 per condition). **c** ELISA analysis of TNF-α and IL-6 levels in the serum of WT or *Zc3hav1*^-/-^ mice 12 h after i.p. injection of HSV-1 (2 × 10^7^ PFU per mouse) (PBS, *n* = 2; HSV-1, *n* = 5 per condition). **d** ELISA analysis of IL-6 serum levels in WT or *Zc3hav1*^-/-^ mice 4 h after i.p. injection of DMXAA (23 mg/kg) (PBS, *n* = 2; DMXAA, *n* = 6 per condition). **e** RT-PCR analysis of *Il6* mRNA levels in lung, spleen and liver tissues from WT or *Zc3hav1*^-/-^ mice 4 h after i.p. injection of DMXAA (23 mg/kg) (PBS, *n* = 2; DMXAA, *n* = 4 or 6 per condition). **f**, **g** ELISA analysis of IL-6 levels in the serum of WT or *Zc3hav1*^-/-^ mice treated with 750 nM H-151 per mouse for 1 h and then stimulated with HSV-1 (2 × 10^7^ PFU per mouse) for 12 h or DMXAA (23 mg/kg) for 4 h (PBS, *n* = 2 or 3; HSV-1, *n* = 6; DMXAA, *n* = 5). All data are represented as means ± SD. One representative result from three independent experiments was shown. Statistical significance was determined by an unpaired two-tailed Student’s *t* test in (**a**–**e**) and ANOVA test in (**f**, **g**): * *P* ≤ 0.05, ** *P* ≤ 0.01, *** *P* ≤ 0.001. ns, not significant (*P* > 0.05).

To evaluate the therapeutic potential of ZC3HAV1 in the STING pathway in vivo, we infected *Zc3hav1*^-/-^ mice with HSV-1. *Ifna4* mRNA expression in the spleens of *Zc3hav1*^*-/-*^ mice after HSV-1 infection was inhibited (Fig. 5a). The plaque assay revealed that the replication of HSV-1 in both the spleen and lung tissues was increased by *Zc3hav1* deficiency (Fig. 5b). The release of the proinflammatory cytokines TNF-α and IL-6 in the serum was significantly impaired in HSV-1-infected *Zc3hav1*^-/-^ mice (Fig. 5c).

We further investigated the physiological and pathological relevance of ZC3HAV1 function in the context of STING activation in vivo. The IL-6 concentration in the serum of *Zc3hav1*^-/-^ mice was significantly lower than that in the serum of the control mice after intraperitoneal (i.p.) injection of DMXAA (Fig. 5d). Moreover, *Il6* mRNA expression in the lung, spleen and liver was markedly decreased in *Zc3hav1*^-/-^ mice (Fig. 5e). Furthermore, the STING inhibitor H-151 reduced IL-6 secretion and counteracted the pronounced differences in IL-6 secretion between *Zc3hav1*^-/-^ mice and the control mice after infection with HSV-1 or injection of DMXAA (Fig. 5f, g). These in vivo data agree with the in vitro data obtained in macrophages, which together demonstrated that ZC3HAV1 can induce STING-dependent inflammation by increasing the systemic cytokine responses.

## Discussion

The cGAS-STING pathway is involved primarily in the response to microbial infection. Hence, the activation of this signaling pathway could be explored as a viable strategy to combat infections. In this study, we showed that ZC3HAV1 was crucial for the activation of the cGAS-STING pathway through the targeting of STING. On the one hand, ZC3HAV1 markedly promoted STING-dependent IRF3 and NF-κB pathway activation in response to stimulation with HSV-1, ISD, cGAMP or DMXAA. On the other hand, ZC3HAV1 potentiated HSV-1-, ISD-, cGAMP- or DMXAA-induced systemic cytokine responses. We demonstrated that ZC3HAV1 facilitated STING activation by associating with STING and promoting its palmitoylation, oligomerization, and translocation from the ER to the Golgi. These results provide insights into the mechanism of STING pathway activation.

Oligomerization of STING constitutes a vital prerequisite for STING activation^38^. Except being enhanced by palmitoylation at Cys88 and Cys91, Cys148 residues in the connector helix of STING can form a disulfide bond and thereby stabilize oligomerization^39^. Small-molecule agonists, such as Compound 53 (C53), promote the oligomerization by binding to a cryptic pocket in the STING transmembrane domain^40^. In addition, a genome-wide CRISPR-Cas9 screen identified sulfated glycosaminoglycans (sGAGs) that bind to STING and induce STING oligomerization^41^. Various other factors have been proposed to negatively regulate STING oligomerization. Recently, UNC13D, which is associated with familial hemophagocytic lymphohistiocytosis (FHL3), was reported to colocalize and directly interact with STING and inhibit STING oligomerization^42^. The African swine fever virus (ASFV) H240R protein (pH240R) interacts with the N-terminal transmembrane domain of STING and inhibits its oligomerization, suggesting that pH240R is a potent antagonist of the STING pathway^43^. Our results indicated that ZC3HAV1 interacts with STING in its resting state and enhances STING oligomerization. Upon STING ligand engagement, extensive conformational changes in STING likely facilitate its interaction with ZC3HAV1, and the combined ZC3HAV1 may further stabilize the dimeric form of STING and consequently promote STING oligomerization. However, the underlying mechanism remains to be further elucidated.

Numerous studies have proven the direct role of ZC3HAV1 in protecting against RNA viruses, and several studies have revealed the involvement of ZC3HAV1 in the defense against DNA viruses. For example, ZC3HAV1 targets HCMV *UL4-UL6* mRNA for degradation, thereby inhibiting the replication of HCMV^44^. The C16 protein of the Ankara vaccine virus antagonizes ZC3HAV1, whereas ZC3HAV1 interferes with the assembly of infectious MVA (modified vaccinia virus Ankara with C16 disruption) virions^45^. In addition, ZC3HAV1 restricts HBV as HBV uses pregenomic (pg) RNA precursors as templates for its replication, and ZC3HAV1 downregulates HBV pgRNA^46^. ZC3HAV1 employs distinct mechanisms to exhibit antiviral activity. ZAPS induces the production of IFNs-I in response to CpG-enriched IAV infection and ZAPS associates with RIG-I and enhances the ATPase activity and oligomerization of RIG-I, indicating that ZC3HAV1 utilizes another mode of action to sense RNA viruses^34^. Here, we demonstrated that ZC3HAV1 facilitates the DNA virus HSV-1 infection-induced STING signaling by associating with STING and promoting its oligomerization and ER-to-Golgi trafficking. Thus, our study explored a new role for ZC3HAV1 in activating the cGAS-STING pathway against DNA viruses.

Although the transient inflammatory response is crucial for combatting pathogens, accumulating evidence has revealed a pathogenic role for sustained and chronic STING activation in a range of complex inflammatory and autoimmune diseases^47^. The STING V155M mutation results in constitutive STING activation, leading to chronic inflammation such as pulmonary inflammation^48^. The inability to eliminate self-DNA effectively may also be a key contributor to inflammatory diseases. For example, *Dnase2*^*-/-*^ mice develop a polyarthritis and induce high levels of TNF-α and IL-6 in affected joints, and blockade of TNF-α or IL-6 has a therapeutic effect on arthritis in *Dnase2*^*-/-*^ mice^49^. In addition, depletion of STING reverses Dnase2-dependent embryonic lethality and polyarthritis because cytosolic DNA-driven cytokine production is eliminated^50^. Thus, designing drugs that repress STING activation may achieve anti-inflammatory effects. For instance, a selective small-molecule antagonist, H-151, reduces STING-mediated inflammatory cytokine production and pathological features by blocking the palmitoylation of STING at Cys91^51^. In this study, we emphasized the role of ZC3HAV1 in STING-induced inflammation and showed that *Zc3hav1* deficiency protects mice against HSV-1 infection- and DMXAA-induced systemic cytokine responses. Taken together, our findings have revealed the proinflammatory effect of ZC3HAV1, which provides a potential ZC3HAV1-STING-targeting strategy for the clinical management of STING-dependent inflammatory diseases.

## Materials and methods

### Mice

*Zc3hav1*-deficient (*Zc3hav1*^-/-^) mice (T014253) were obtained from GemPharmatech Co.Ltd. *Sting*-deficient (*Sting*^*-/-*^) mice (025805) were obtained from the Jackson Laboratory. All animals were used at 6–10 weeks of age, and both male and female mice were included. All the animals were housed at specific pathogen-free (SPF) levels with 40–70% humidity and daily cycles of 12 h of light at 23 °C and 12 h of dark at 21 °C. All animal experiments were performed in accordance with the National Institutes of Health Guide for the Care and Use of Laboratory Animals, with the approval of the Scientific Investigation Board of the School of Basic Medical Science, Shandong University, Jinan, Shandong Province, China.

### Cell culture

To obtain mouse primary peritoneal macrophages, C57BL/6J mice (6–10 weeks old) were injected intraperitoneally (i.p.) with 3% Brewer’s thioglycollate broth. Three days later, the peritoneal exudate cells (PECs) were harvested and incubated. Two hours later, nonadherent cells were discarded, and adherent monolayer cells were used as PMs. THP-1 (TCHu57) and human embryonic kidney (HEK293T, GNHu17) cells were obtained from the National Collection of Authenticated Cell Cultures. Phorbol myristate acetate-activated THP-1 cells were used as human macrophages. MEFs were generated from female mice that were identified as pregnant for 13–14 days. Briefly, the fetal viscera, head, and limbs were excised from the embryos, and the remaining embryonic tissues were minced and incubated with 0.25% trypsin-EDTA for 30 min at 37 °C. The MEFs were cultured and expanded for subsequent experiments. The cells were cultured at 37 °C under 5% CO_2_ in Dulbecco’s modified Eagle’s medium (DMEM) supplemented with 10% fetal calf serum (Procell), 100 U mL^-1^ penicillin, and 100 μg mL^-1^ streptomycin.

### Reagents and antibodies

2′3′-cGAMP (tlrl-nacga23-1), DMXAA (tlrl-dmx), ODN 1826 (tlrl-1826), Pam3CSK4 (tlrl-pms), R848 (tlrl-r848) and ISD (tlrl-isdn) were obtained from InvivoGen. LPS (*Escherichia coli*, O111:B4, L4130) and poly(I:C) (P1530) were obtained from Sigma Aldrich. HSV-1 and VSV were obtained from X. Cao (Second Military Medical University, Shanghai, China). H-151 (HY-112693) was obtained from MedChemExpress. Stimulants were used at concentrations as follows: ISD, 5 μg/mL; cGAMP, 3 μg/mL; DMXAA, 150 μg/mL; LPS, 200 ng/mL; Pam3CSK4, 1 μg/mL; poly(I:C), 20 μg/mL; R848, 10 μg/mL; ODN 1826, 2 μΜ; H-151, 1 μM; VSV, infection at a multiplicity of infection (MOI) of 1. The protein A/G agarose (sc-2003) used for immunoprecipitation (IP) was from Santa Cruz Biotechnology. Horseradish peroxidase-conjugated AffiniPure goat anti-rabbit IgG (H + L) (SA00001-2, 1:5000) and an anti-β-actin (66009-1-Ig, 1:2000) antibody were obtained from Proteintech; anti-mouse IgG (AC011, 1:200 for IP) was obtained from ABclonal; anti-rabbit IgG (2729, 1:400 for IP), anti-p-IκBα (Ser32, 14D4, 2859, 1:1000), anti-p-P65 (Ser536, 93H1, 3033, 1:1000), anti-P65 (D14E12, 8242, 1:1000), anti-STING (D1V5L, 50494, 1:1000 for immunoblot,1:400 for IP), anti-p-IRF3 (Ser396, 4D4G, 4947, 1:1000), anti-IRF3 (D83B9, 4302, 1:1000), anti-p-TBK1 (Ser172, D52C2, 5483S, 1:1000) and anti-TBK1 (3013, 1:1000) antibodies were obtained from Cell Signaling Technology; anti-ZC3HAV1 (16820-1-AP, 1:1000) and anti-STING (19851-1-AP, 1:200 for immunofluorescence) antibodies were obtained Proteintech. Anti-HA (HA-7, H3663, 1:1000 for immunoblot), anti-Myc (9E10, M4439, 1:1000 for immunoblot), and anti-Flag (M2, F1804, 1:1000 for immunoblot, 1:400 for IP) antibodies were purchased from Sigma Aldrich. Anti-HA (CB051, TA180128-1, 1:400 for IP) and anti-Myc (9E10, TA1501211, 1:400 for IP and 1:100 for immunofluorescence) antibodies were purchased from Origene. Biotin-cGAMP (C157) was obtained from BioLog Life Science Institute (C 157-001). An anti-GM130 (sc-55591) antibody was purchased from Santa Cruz Biotechnology. An anti-Calnexin (ab22595) antibody was purchased from Abcam. Goat anti-rabbit IgG Alexa Fluor 633 (A-21071) and rabbit anti-mouse IgG Alexa Fluor 488 (A-11059) were obtained from Thermo Fisher Scientific.

### Immunoblot and IP analysis

For immunoblot analysis, the cells were lysed with Pierce^TM^ RIPA Buffer (Thermo Fisher Scientific, 89901) supplemented with a protease inhibitor cocktail (Sigma Aldrich, P8340), and the protein concentrations in the extracts were measured with a Pierce™ BCA protein assay (Thermo Fisher Scientific, 23228). Equal amounts of extracts were separated by SDS‒PAGE, and then transferred onto polyvinylidene fluoride (PVDF) membranes for immunoblot analysis. For IP analysis, whole-cell lysates were lysed in IP buffer containing 1.0% Nonidet P 40, 50 mM Tris-HCl (pH 7.4), 50 mM EDTA, 150 mM NaCl, and a protease inhibitor cocktail. After 15 min of centrifugation at 12,000 × *g* at 4 °C, the supernatants were collected and incubated with protein G Plus-Agarose IP reagent together with a specific antibody overnight. The beads were subsequently washed five times with IP buffer. The immunoprecipitates were eluted by boiling with 1% SDS sample buffer. The original blot images were presented Supplementary Fig. 3.

### In vitro pull-down analysis

For in vitro pull-down assay analysis of biotin-cGAMP binding to STING, whole-cell lysates were lysed in IP buffer. After 15 min of centrifugation at 12,000 × *g* at 4 °C, the supernatants were collected and incubated with biotin-cGAMP for 2 h at 4 °C and then enriched by the addition of Pierce™ Streptavidin Agarose (Thermo Fisher Scientific, 20347) for 4 h at 4  °C. The samples were subsequently washed five times with IP buffer. The immunoprecipitates were eluted by boiling with 1% SDS sample buffer.

### ELISA and real-time PCR

Secreted mouse TNF-α (cat. 1217203) and mouse IL-6 (cat. 1210602) were measured with ELISA kits (Dakewe Biotech Company Ltd.). Intracellular cGAMP levels were measured with ELISA kits (Cayman, 501700) according to the manufacturer’s instructions. Total RNA was extracted with an RNA fast 200 RNA Extraction Kit (Fastagen, 220011). The RNA (500 ng) was reverse transcribed with reverse transcriptase (Vazyme, HiScript III RT SuperMix for qPCR ( + gDNA wiper), R323-01). Quantitative real-time PCR analysis was carried out with the Applied Biosystems StepOnePlus Real-Time PCR System and SYBR RT-PCR kits (Vazyme, ChamQ Universal SYBR qPCR Master Mix, Q711-03). The data were normalized to β-actin expression in each sample. The sequences of PCR primers are listed in Supplementary Table 1.

### RNA interference assay

For transient silencing of endogenous ZC3HAV1, siRNAs were synthesized as follows: si*Zc3hav1*-1 sense: 5′-CCGAUCGCUUUGUGCUAUU-3′, and antisense: 5′-AAUAGCACAAAGCGAUCGG-3′; si*Zc3hav1*-2 sense: 5′-CCUGCUGAGUAUUCCCUAU-3′, and antisense: 5′-AUAGGGAAUACUCAGCAGG-3′. The mixture of three siRNAs targeting mouse *Rig-i* was designated as si*Rig-i*, and the sequences of the siRNAs were as follows: si*Rig-i*-1sense: 5′-GGCCACAGUUGAUCCAAAU-3′, and antisense: 5′-AUUUGGAUCAACUGUGGCCTT-3′; si*Rig-i*-2 sense: 5′-CCAGCGGAGAUAACAAUAU-3′, and antisense: 5′-AUAUUGUUAUCUCCGCUGGTT-3′; and si*Rig-i*-3 sense: 5′-GGACUUCGAACACGUUUAA-3′, and antisense: 5′-UUAAACGUGUUCGAAGUCCTT-3′. The control sequence was 5′-UUCUCCGAACGUGUCACGU-3′. These siRNA duplexes were transfected into mouse PMs with INTERFERin reagents (PolyPlus, 409-10) according to the manufacturer’s instructions.

### Plasmids and transfection

The ZAPS expression plasmid (CH892071) was purchased from WZ Bioscience, Inc., and then a Flag-tag was added. The ZAPL expression plasmid (YZ-B021001) was generated by Changsha Youze Biotechnology Co., Ltd. The tagged STING plasmids were described previously^52^. All the constructs were confirmed by DNA sequencing. The plasmids were transiently transfected into HEK293T cells with Lipofectamine 2000 reagent (Invitrogen, 11668019) according to the manufacturer’s instructions.

### Immunofluorescence staining and confocal microscopy

MEFs from WT mice or *Zc3hav1*^-/-^ mice were plated on coverslips in 24-well plates. The cells on the coverslips were fixed with Immunol Staining Fix Solution (Beyotime, P0098), permeabilized with 0.5% Triton-X 100 in PBS, blocked in 3% BSA for 1 h, and then incubated with primary antibodies (anti-Myc, anti-GM130 or anti-Calnexin) for 8 h at 4 °C. The coverslips were rinsed with PBS twice, and the secondary antibody (Alexa Fluor 633 or Alexa Fluor 488) was added directly to the center of the coverslips and incubated for 1 h. The nuclei were stained with DAPI (Solarbio, C0065) for 3 min. Then, the cells were subjected to microscopy analysis with a laser confocal microscope (Zeiss Laser confocal microscope LSM980).

### Native PAGE analysis of protein aggregation

PMs were lysed in lysis buffer containing 1.0% Nonidet P- 40, 10.0% 0.5 M Tris-HCl (pH 7.5), 10.0% 0.5 M EDTA, 150 mM NaCl, and a protease inhibitor cocktail on ice for 10 min. The cells were then scraped and incubated gently on ice for 10 min. After 15 min of centrifugation at 12,000 × *g* at 4 °C, the protein concentrations in the extracts were measured and 6×Native-PAGE loading buffer (70.0% 4×Tris-HCl/SDS (pH 6.8), 30.0% glycerol, 1.0% sodium deoxycholate and an appropriate amount of bromophenol blue) was added. The 7% Native Gels (No SDS) with Native-PAGE running buffer (with the cathode buffer containing 0.303% Tris base, 1.44% glycine and 0.2% sodium deoxycholate and the anode buffer containing no sodium deoxycholate) were prerun at 45 mA for 30 min on ice, after which the samples were loaded onto the gels, which were run on ice at 25 mA for 6 h. The proteins were transferred to a 0.45 μm PVDF membrane at 300 mA for 90 min or at 30 V overnight on ice for immunoblot analysis.

### STING palmitoylation by click chemistry

PMs were incubated with 100 μM 15-azido-pentadecanoic acid (NEW RESEARCH BIOSCIENCES, Y-AJS-24014) for 2 h at 37 °C and then infected with HSV-1 for 4 h. The cells were washed twice in PBS, and then lysed on ice in 500 μL of Pierce^TM^ RIPA Buffer supplemented with a protease inhibitor cocktail. The lysate was incubated with 2 mM CuSO_4_ (MACKUN, C805782), 200 μM TBTA ligands (MACKUN, T823729), 200 μM biotin-alkyne (Aladdin, B171422) and  2 mM TCEP (MACKUN, T819166) for 8 h at 4 °C. After precipitation with cold methanol, chloroform and double distilled water, the protein precipitates were dissolved in IP buffer containing 0.5% SDS. The proteins were enriched by the addition of Pierce™ Streptavidin Agarose for 8 h at 4 °C. The samples were subsequently washed five times with IP buffer containing 0.5% SDS and eluted by boiling with 1% SDS sample buffer.

### Plaque assay

Vero cells were plated as confluent monolayers in 12-well plates. The medium was removed and the wells were washed with DMEM without FBS. Then, 500 μL of serially diluted cell culture supernatant or homogenized tissue lysates was added to the wells, followed by incubation for 1 h at 37 °C, in a 5% CO_2_ incubator. After viral adsorption, the supernatant was removed from the wells, and the cells were overlaid with overlay medium (DMEM containing 1% methylcellulose). Twenty-four or 48 h post infection, the cells were fixed with 4% paraformaldehyde for 15 min, and stained with 1% crystal violet for 20 min to count the plaques for the calculation of viral titers.

### Viral infection in vivo

For WT and *Zc3hav1*^-/-^ mice, 6–8-week-old and sex-matched littermates were infected by intraperitoneal injection of 2 × 10^7^ plaque-forming units (PFU) HSV-1 for 12 h; the serum of the mice was collected for ELISA analysis, and spleen tissues were obtained for quantitative PCR. WT and *Zc3hav1*^-/-^ mice, 6–8-week-old and sex-matched littermates, were infected by intraperitoneal injection of HSV-1 (2 × 10^7^ PFU per mouse) for 48 h, after which spleen and lung tissues were collected for plaque assays.

### In vivo DMXAA model

WT or *Zc3hav1*^-/-^ mice (males, 16–18 g) were intraperitoneally injected with 23 mg/kg DMXAA. After 4 h, the serum of the mice was collected for ELISA analysis of IL-6, and then the mice were euthanized to obtain liver, spleen and lung tissues for quantitative PCR.

### Statistics and reproducibility

The colocalization of Immunofluorescence and the quantification of immunoblot were analyzed by ImageJ. Statistical significance between groups was determined by two-tailed Student’s *t* test or ANOVA test by GraphPad Prism 8.0.2 software. Sample size and replicates are stated in corresponding figure legends. *P* ≤ 0.05 were considered statistically significant. *P ≤ 0.05, **P ≤ 0.01, and ***P ≤ 0.001. All quantitative measurements were tested for a normal distribution.

## Supplementary information

Supplementary Information

Description of Additional Supplementary File

Supplementary Data 1

## Data Availability

All data supporting the findings of this study are available within the article and its Supplementary Information. The source data behind the graphs in the paper is available in Supplementary Data 1. All other data are available from the corresponding author on reasonable request.

## References

[1] Sun, L., Wu, J., Du, F., Chen, X. & Chen, Z. J. Cyclic GMP-AMP synthase is a cytosolic DNA sensor that activates the type I interferon pathway. *Science* **339**, 786–791 (2013). 23258413 10.1126/science.1232458 PMC3863629

[2] Ablasser, A. et al. cGAS produces a 2’-5’-linked cyclic dinucleotide second messenger that activates STING. *Nature* **498**, 380–384 (2013). 23722158 10.1038/nature12306 PMC4143541

[3] Ishikawa, H. & Barber, G. N. STING is an endoplasmic reticulum adaptor that facilitates innate immune signalling. *Nature* **455**, 674–678 (2008). 18724357 10.1038/nature07317 PMC2804933

[4] Zhong, B. et al. The adaptor protein MITA links virus-sensing receptors to IRF3 transcription factor activation. *Immunity* **29**, 538–550 (2008). 18818105 10.1016/j.immuni.2008.09.003

[5] Sun, W. et al. ERIS, an endoplasmic reticulum IFN stimulator, activates innate immune signaling through dimerization. *Proc. Natl Acad. Sci. USA* **106**, 8653–8658 (2009). 19433799 10.1073/pnas.0900850106 PMC2689030

[6] Jin, L. et al. MPYS, a novel membrane tetraspanner, is associated with major histocompatibility complex class II and mediates transduction of apoptotic signals. *Mol. Cell Biol.* **28**, 5014–5026 (2008). 18559423 10.1128/MCB.00640-08 PMC2519703

[7] Wu, J. et al. Cyclic GMP-AMP is an endogenous second messenger in innate immune signaling by cytosolic DNA. *Science* **339**, 826–830 (2013). 23258412 10.1126/science.1229963 PMC3855410

[8] Ishii, K. J. et al. TANK-binding kinase-1 delineates innate and adaptive immune responses to DNA vaccines. *Nature* **451**, 725–729 (2008). 18256672 10.1038/nature06537

[9] Zhang, X., Bai, X. C. & Chen, Z. J. Structures and Mechanisms in the cGAS-STING Innate Immunity Pathway. *Immunity* **53**, 43–53 (2020). 32668227 10.1016/j.immuni.2020.05.013

[10] Darnell, J. J., Kerr, I. M. & Stark, G. R. Jak-STAT pathways and transcriptional activation in response to IFNs and other extracellular signaling proteins. *Science* **264**, 1415–1421 (1994). 8197455 10.1126/science.8197455

[11] Dunphy, G. et al. Non-canonical Activation of the DNA Sensing Adaptor STING by ATM and IFI16 Mediates NF-kappaB Signaling after Nuclear DNA Damage. *Mol. Cell* **71**, 745–760.e5 (2018). 30193098 10.1016/j.molcel.2018.07.034 PMC6127031

[12] Hou, Y. et al. Non-canonical NF-kappaB Antagonizes STING Sensor-Mediated DNA Sensing in Radiotherapy. *Immunity* **49**, 490–503.e4 (2018). 30170810 10.1016/j.immuni.2018.07.008 PMC6775781

[13] Mulero, M. C., Huxford, T. & Ghosh, G. NF-kappaB, IkappaB, and IKK: integral components of immune system signaling. *Adv. Exp. Med. Biol.* **1172**, 207–226 (2019). 31628658 10.1007/978-981-13-9367-9_10

[14] Tsuchida, T. et al. The ubiquitin ligase TRIM56 regulates innate immune responses to intracellular double-stranded DNA. *Immunity* **33**, 765–776 (2010). 21074459 10.1016/j.immuni.2010.10.013

[15] Sun, M. S. et al. TMED2 potentiates cellular IFN responses to DNA viruses by reinforcing MITA dimerization and facilitating its trafficking. *Cell Rep.* **25**, 3086–3098.e3 (2018). 30540941 10.1016/j.celrep.2018.11.048

[16] Wang, X. et al. STING requires the adaptor TRIF to trigger innate immune responses to microbial infection. *Cell Host Microbe* **20**, 329–341 (2016). 27631700 10.1016/j.chom.2016.08.002 PMC5026396

[17] Zou, H. M., et al. Human Cytomegalovirus Protein UL94 Targets MITA to evade the antiviral immune response. *J. Virol.* **94**, 10–1128 (2020). 10.1128/JVI.00022-20 PMC7307088 32238587

[18] MacDonald, M. R., Machlin, E. S., Albin, O. R. & Levy, D. E. The zinc finger antiviral protein acts synergistically with an interferon-induced factor for maximal activity against alphaviruses. *J Virol* **81**, 13509–13518 (2007). 17928353 10.1128/JVI.00402-07 PMC2168828

[19] Gao, G., Guo, X. & Goff, S. P. Inhibition of retroviral RNA production by ZAP, a CCCH-type zinc finger protein. *Science* **297**, 1703–1706 (2002). 12215647 10.1126/science.1074276

[20] Wang, N. et al. Viral induction of the zinc finger antiviral protein is IRF3-dependent but NF-kappaB-independent. *J. Biol. Chem.* **285**, 6080–6090 (2010). 20048147 10.1074/jbc.M109.054486 PMC2825402

[21] Muller, S. et al. Inhibition of filovirus replication by the zinc finger antiviral protein. *J. Virol.* **81**, 2391–2400 (2007). 17182693 10.1128/JVI.01601-06 PMC1865956

[22] Liu, C. H., Zhou, L., Chen, G. & Krug, R. M. Battle between influenza A virus and a newly identified antiviral activity of the PARP-containing ZAPL protein. *Proc. Natl Acad. Sci. USA* **112**, 14048–14053 (2015). 26504237 10.1073/pnas.1509745112 PMC4653199

[23] Bick, M. J. et al. Expression of the zinc-finger antiviral protein inhibits alphavirus replication. *J. Virol.* **77**, 11555–11562 (2003). 14557641 10.1128/JVI.77.21.11555-11562.2003 PMC229374

[24] Zhu, Y. et al. Zinc-finger antiviral protein inhibits HIV-1 infection by selectively targeting multiply spliced viral mRNAs for degradation. *Proc. Natl Acad. Sci. USA* **108**, 15834–15839 (2011). 21876179 10.1073/pnas.1101676108 PMC3179061

[25] Guo, X., Carroll, J. W., Macdonald, M. R., Goff, S. P. & Gao, G. The zinc finger antiviral protein directly binds to specific viral mRNAs through the CCCH zinc finger motifs. *J. Virol.* **78**, 12781–12787 (2004). 15542630 10.1128/JVI.78.23.12781-12787.2004 PMC525010

[26] Kerns, J. A., Emerman, M. & Malik, H. S. Positive selection and increased antiviral activity associated with the PARP-containing isoform of human zinc-finger antiviral protein. *PLoS Genet.* **4**, e21 (2008). 18225958 10.1371/journal.pgen.0040021 PMC2213710

[27] Luo, X. et al. Molecular mechanism of RNA recognition by Zinc-Finger antiviral protein. *Cell Rep.* **30**, 46–52.e4 (2020). 31914396 10.1016/j.celrep.2019.11.116

[28] Takata, M. A. et al. CG dinucleotide suppression enables antiviral defence targeting non-self RNA. *Nature* **550**, 124–127 (2017). 28953888 10.1038/nature24039 PMC6592701

[29] Zhu, Y., Wang, X., Goff, S. P. & Gao, G. Translational repression precedes and is required for ZAP-mediated mRNA decay. *EMBO J.* **31**, 4236–4246 (2012). 23023399 10.1038/emboj.2012.271 PMC3492732

[30] Chen, S. et al. Structure of N-terminal domain of ZAP indicates how a zinc-finger protein recognizes complex RNA. *Nat. Struct. Mol. Biol.* **19**, 430–435 (2012). 22407013 10.1038/nsmb.2243

[31] Goncalves-Carneiro, D. et al. Rational attenuation of RNA viruses with zinc finger antiviral protein. *Nat. Microbiol.* **7**, 1558–1567 (2022). 36075961 10.1038/s41564-022-01223-8 PMC9519448

[32] Meagher, J. L. et al. Structure of the zinc-finger antiviral protein in complex with RNA reveals a mechanism for selective targeting of CG-rich viral sequences. *Proc. Natl Acad. Sci. USA* **116**, 24303–24309 (2019). 31719195 10.1073/pnas.1913232116 PMC6883784

[33] Glasker, S., Toller, M. & Kummerer, B. M. The alternate triad motif of the poly(ADP-ribose) polymerase-like domain of the human zinc finger antiviral protein is essential for its antiviral activity. *J. Gen. Virol.* **95**, 816–822 (2014). 24457973 10.1099/vir.0.060988-0

[34] Hayakawa, S. et al. ZAPS is a potent stimulator of signaling mediated by the RNA helicase RIG-I during antiviral responses. *Nat. Immunol.* **12**, 37–44 (2011). 21102435 10.1038/ni.1963

[35] Schwerk, J. et al. RNA-binding protein isoforms ZAP-S and ZAP-L have distinct antiviral and immune resolution functions. *Nat. Immunol.* **20**, 1610–1620 (2019). 31740798 10.1038/s41590-019-0527-6 PMC7240801

[36] Zhang, B. C. et al. STEEP mediates STING ER exit and activation of signaling. *Nat. Immunol.* **21**, 868–879 (2020). 32690950 10.1038/s41590-020-0730-5 PMC7610351

[37] Mukai, K. et al. Activation of STING requires palmitoylation at the Golgi. *Nat. Commun.* **7**, 11932 (2016). 27324217 10.1038/ncomms11932 PMC4919521

[38] Shang, G., Zhang, C., Chen, Z. J., Bai, X. C. & Zhang, X. Cryo-EM structures of STING reveal its mechanism of activation by cyclic GMP-AMP. *Nature* **567**, 389–393 (2019). 30842659 10.1038/s41586-019-0998-5 PMC6859894

[39] Ergun, S. L., Fernandez, D., Weiss, T. M. & Li, L. STING polymer structure reveals mechanisms for activation, hyperactivation, and inhibition. *Cell* **178**, 290–301.e10 (2019). 31230712 10.1016/j.cell.2019.05.036

[40] Lu, D. et al. Activation of STING by targeting a pocket in the transmembrane domain. *Nature* **604**, 557–562 (2022). 35388221 10.1038/s41586-022-04559-7 PMC9098198

[41] Fang, R. et al. Golgi apparatus-synthesized sulfated glycosaminoglycans mediate polymerization and activation of the cGAMP sensor STING. *Immunity* **54**, 962–975.e8 (2021). 33857420 10.1016/j.immuni.2021.03.011

[42] Song, P. et al. UNC13D inhibits STING signaling by attenuating its oligomerization on the endoplasmic reticulum. *EMBO Rep.* **23**, e55099 (2022). 36125406 10.15252/embr.202255099 PMC9638857

[43] Ye, G. et al. African Swine Fever Virus H240R protein inhibits the production of Type I Interferon through disrupting the oligomerization of STING. *J. Virol*. **97**, e0057723 (2023). 10.1128/jvi.00577-23 PMC10537660 37199611

[44] Gonzalez-Perez, A. C. et al. The Zinc Finger Antiviral Protein ZAP Restricts Human Cytomegalovirus and Selectively Binds and Destabilizes Viral UL4/UL5 Transcripts. *mBio*. **12**, 10–1128 (2021). 10.1128/mBio.02683-20 PMC8263000 33947766

[45] Peng, C. et al. Zinc-finger antiviral protein (ZAP) is a restriction factor for replication of modified vaccinia virus Ankara (MVA) in human cells. *PLoS Pathog* **16**, e1008845 (2020). 32866210 10.1371/journal.ppat.1008845 PMC7485971

[46] Mao, R. et al. Inhibition of hepatitis B virus replication by the host zinc finger antiviral protein. *PLoS Pathog* **9**, e1003494 (2013). 23853601 10.1371/journal.ppat.1003494 PMC3708887

[47] Barber, G. N. STING: infection, inflammation and cancer. *Nat. Rev. Immunol.* **15**, 760–770 (2015). 26603901 10.1038/nri3921 PMC5004891

[48] Jeremiah, N. et al. Inherited STING-activating mutation underlies a familial inflammatory syndrome with lupus-like manifestations. *J. Clin. Investig.* **124**, 5516–5520 (2014). 25401470 10.1172/JCI79100 PMC4348945

[49] Kawane, K., Tanaka, H., Kitahara, Y., Shimaoka, S. & Nagata, S. Cytokine-dependent but acquired immunity-independent arthritis caused by DNA escaped from degradation. *Proc. Natl Acad. Sci. USA* **107**, 19432–19437 (2010). 20974942 10.1073/pnas.1010603107 PMC2984163

[50] Ahn, J., Gutman, D., Saijo, S. & Barber, G. N. STING manifests self DNA-dependent inflammatory disease. *Proc. Natl Acad. Sci. USA* **109**, 19386–19391 (2012). 23132945 10.1073/pnas.1215006109 PMC3511090

[51] Haag, S. M. et al. Targeting STING with covalent small-molecule inhibitors. *Nature* **559**, 269–273 (2018). 29973723 10.1038/s41586-018-0287-8

[52] Jia, M. et al. Redox homeostasis maintained by GPX4 facilitates STING activation. *Nat. Immunol.* **21**, 727–735 (2020). 32541831 10.1038/s41590-020-0699-0

