# Supplementary material for: ZC3HAV1 facilitates STING activation and enhances inflammation
Source: Commun Biol. 2024 Oct 30;7:1418. doi: 10.1038/s42003-024-07116-2 (PMC11526107; doi:10.1038/s42003-024-07116-2)
Supplement: Supplementary file 1 — Supplementary Information [file 42003_2024_7116_MOESM1_ESM.pdf]

ZC3HAV1 facilitates STING activation and enhances inflammation

Danhui Qin[1], Hui Song[1], Caiwei Wang[1], Xiaojie Ma[2], Yu Fu[3], Chunyuan Zhao[1], Wei Zhao[1], Lei Zhang[3]*, Weifang Zhang[1]*

[1]Key Laboratory for Experimental Teratology of the Chinese Ministry of Education, and Key Laboratory of Infection and Immunity of Shandong Province, School of Basic Medical Science, Qilu Hospital, Cheeloo College of Medicine, Shandong University, Jinan, Shandong, China

[2]Department of Rheumatology and immunology, Affiliated Hospital of Shandong University of Traditional Chinese Medicine, Jinan, Shandong, China

[3]Department of Orthopedic Surgery, the First Affiliated Hospital of Shandong First Medical University & Shandong Provincial Qianfoshan Hospital, Shandong Key Laboratory of Rheumatic Disease and Translational Medicine, Jinan, Shandong, China

* **Correspondence:** qygkzl1818@163.com (L.Z.), zhangweifang@sdu.edu.cn (W-F. Z.)

Supplementary Information

Supplementary Figure 1

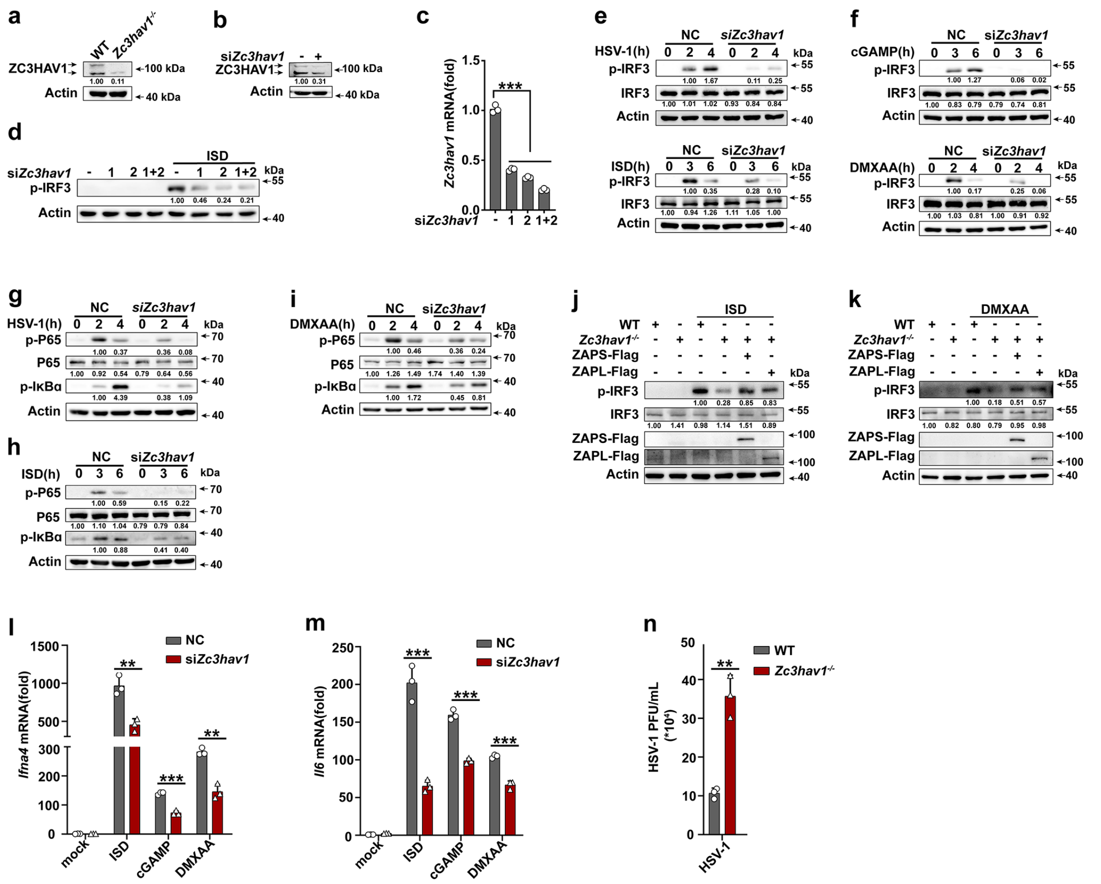

Fig. S1: ZC3HAV1 knockdown inhibits STING signaling.

a Immunoblot analysis of endogenous ZC3HAV1 expression in PMs from WT and *Zc3hav1*$^{-/-}$ mice. **b** Immunoblot analysis of endogenous ZC3HAV1 expression in mouse PMs transfected with control (NC) siRNA or *Zc3hav1* siRNA for 48 h. **c** RT-PCR analysis of *Zc3hav1* mRNA levels in PMs transfected with NC siRNA or *Zc3hav1* siRNA for 48 h. **d** Immunoblot analysis of phosphorylated (p-) IRF3 in mouse PMs transfected with NC siRNA or *Zc3hav1* siRNA for 48 h and subsequently stimulated with ISD. **e, f** Immunoblot analysis of phosphorylated and total IRF3 protein in mouse PMs transfected with NC siRNA or *Zc3hav1* siRNA for 48 h and subsequently stimulated with HSV-1, ISD, cGAMP or DMXAA. **g-i** Immunoblot analysis of the phosphorylation of P65, IκBα and total P65 protein in mouse PMs transfected with NC siRNA or *Zc3hav1* siRNA for 48 h and subsequently stimulated with HSV-1, ISD or DMXAA. **j, k** Immunoblot analysis of phosphorylated and total IRF3 protein in MEFs from WT and *Zc3hav1*$^{-/-}$ mice transfected with the control vector and Flag-ZAPS or Flag-ZAPL and subsequently stimulated with ISD or DMXAA. **l, m** RT-PCR analysis of *Ifna4* and *Il6* mRNA levels in ISD-, cGAMP- or DMXAA-stimulated mouse PMs transfected with NC siRNA or *Zc3hav1* siRNA for 48 h. **n** Plaque assay analysis of the viral titers of HSV-1 in PMs from WT and *Zc3hav1*$^{-/-}$ mice infected with HSV-1. All data are represented as means ± SD. All experiments were repeated at a minimum of three times. Statistical significance was determined by unpaired two-tailed Student's *t* test: ** P ≤ 0.01, and *** P ≤ 0.001.

Supplementary Figure 2

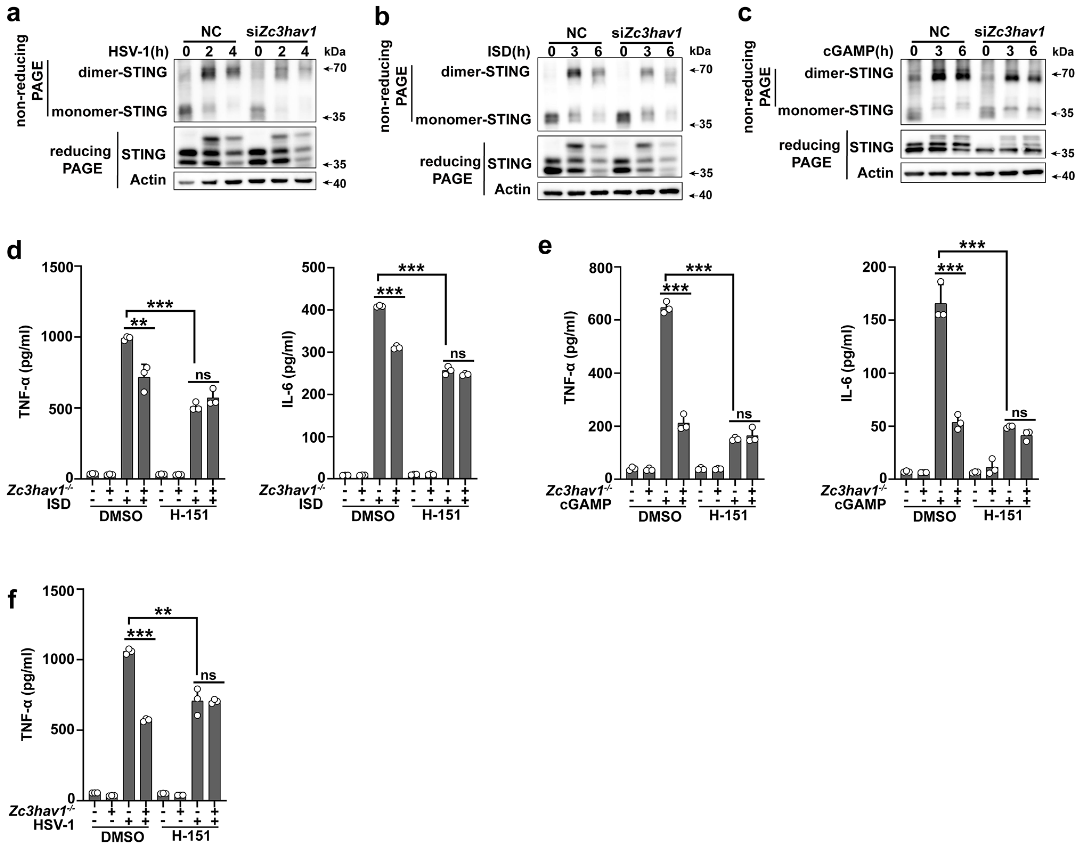

Fig. S2: ZC3HAV1 knockdown suppresses STING activation.

a Native PAGE and immunoblot analysis of the dimerization of endogenous STING in mouse PMs transfected with NC siRNA or *Zc3hav1* siRNA for 48 h and subsequently infected with HSV-1. **b, c** Native PAGE and immunoblot analysis of ISD- or cGAMP-induced dimerization of endogenous STING in mouse PMs transfected with NC siRNA or *Zc3hav1* siRNA for 48 h. **d-f** ELISA analysis of TNF-α and IL-6 secreted from PMs of WT and *Zc3hav1*$^{-/-}$ mice treated with H-151 (for 1 h) and subsequently stimulated with ISD, cGAMP or HSV-1. All data are represented as means ± SD. Similar results were obtained from three independent experiments. Statistical significance was determined by ANOVA test in **d-f**: ** P ≤ 0.01, *** P ≤ 0.001, ns, not significant ($P > 0.05$).

Supplementary Figure 3: Uncropped blot images

Uncropped blot images of Figure 1

Fig.1a

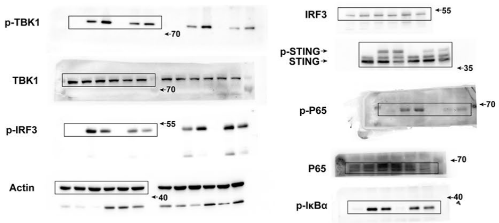

Fig.1b

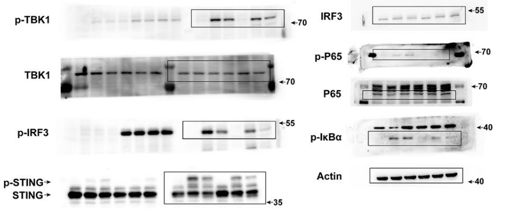

Fig.1c

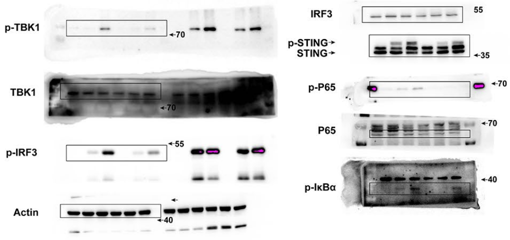

Fig.1d

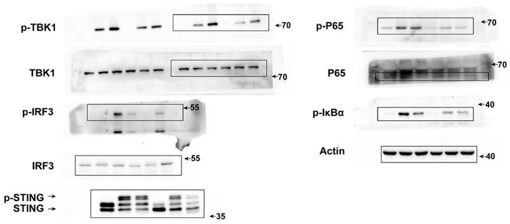

Uncropped blot images of Figure 2

Fig.2b

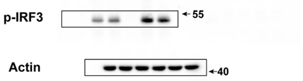

Fig.2c

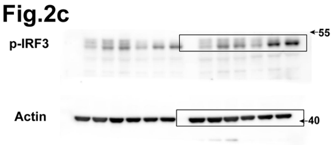

Fig.2d

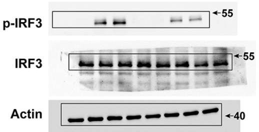

Fig.2e

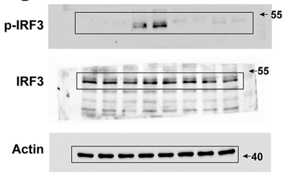

Fig.2h

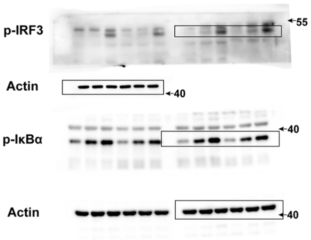

Fig.2i

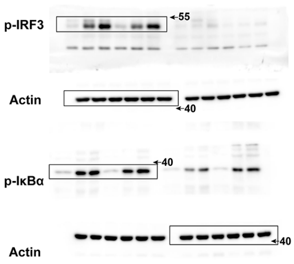

Fig.2j

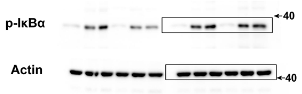

Fig.2k

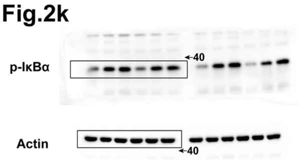

Uncropped blot images of Figure 3

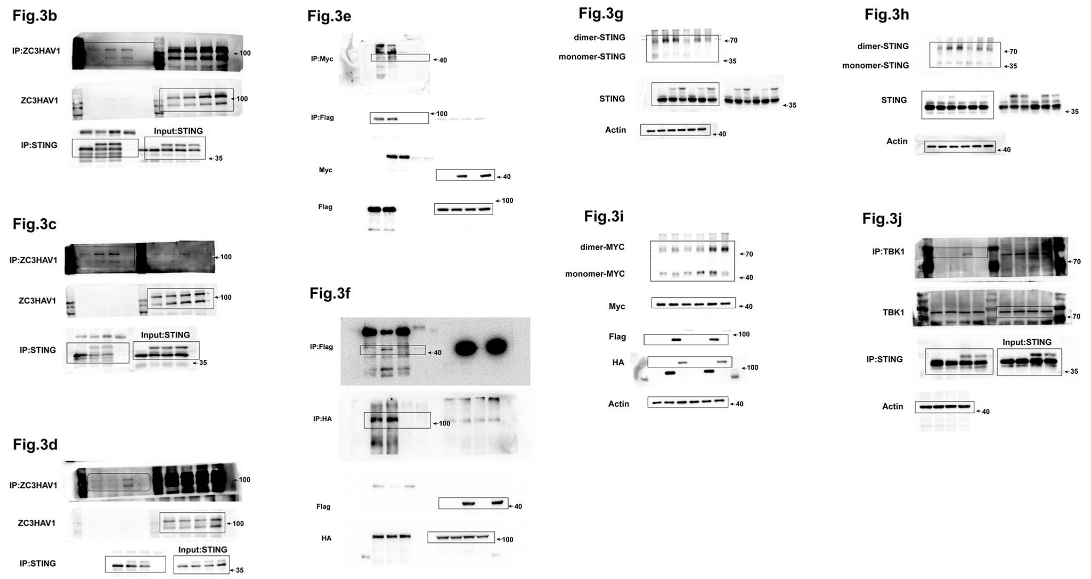

Uncropped blot images of Figure 4

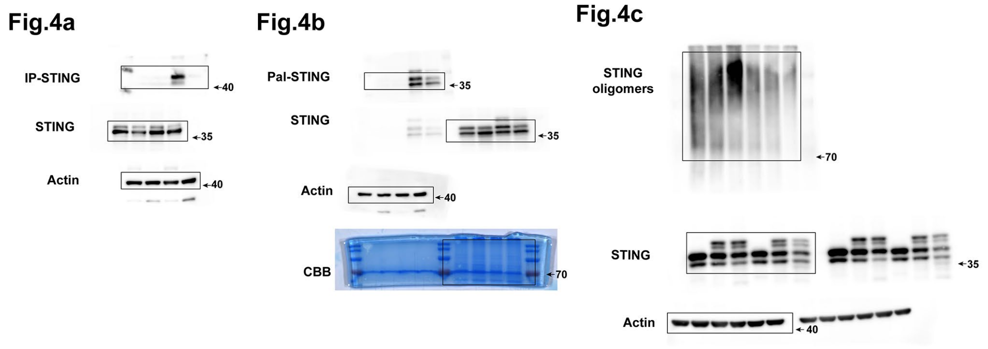

Uncropped blot images of Figure S1

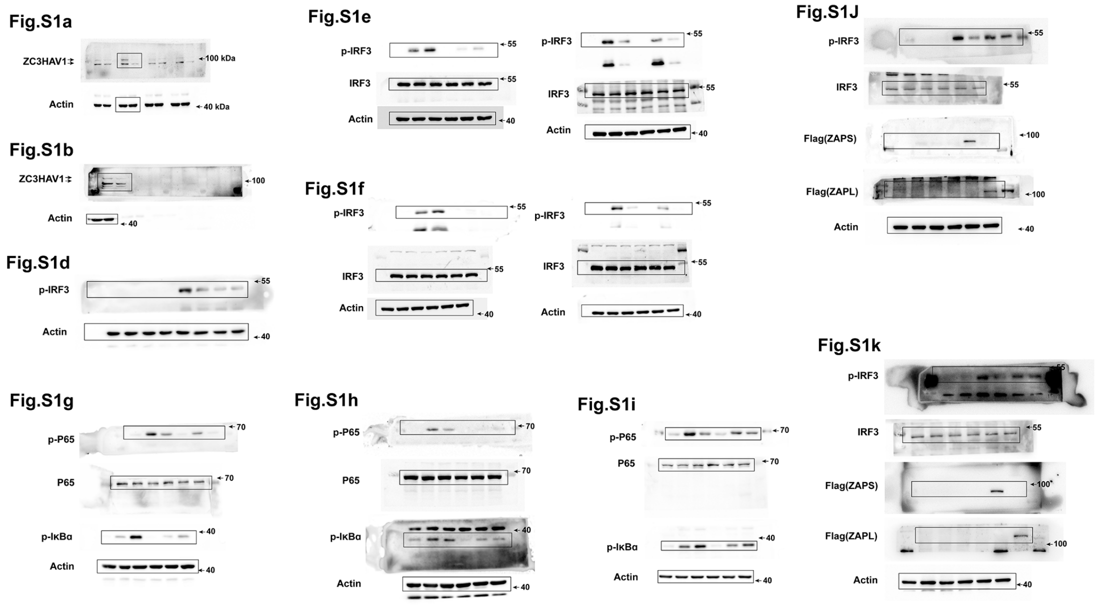

Uncropped blot images of Figure S2

Fig.S2a

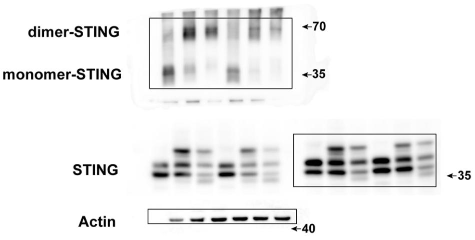

Fig.S2b

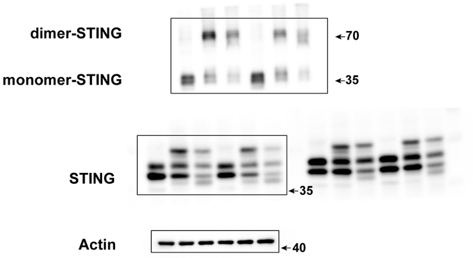

Fig.S2c

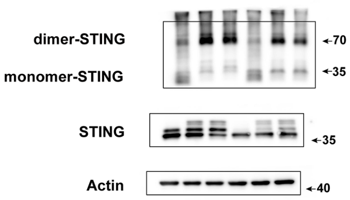

Supplementary Table 1

Table S1. Sequences of PCR primers used in this study

Name	Prime	Sequence
m*Actin*	Forward	5’-TGTTACCAACTGGGACGACA-3’
	Reverse	5’-CTGGGTCATCTTTTCACGGT- 3’
m*Ifna4*	Forward	5′-GACTTGTCTGCTACTTGGAATGC-3′
	Reverse	5′-TTGGTTGAGGAAGAGAGGGCT-3′
m*Tnfa*	Forward	5'-GCCACCACGCTCTTCTGTCT-3'
	Reverse	5'-TGAGGGTCTGGGCCATAGAAC-3'
m*Il6*	Forward	5'-ACAACCACGGCCTTCCCTAC-3'
	Reverse	5'-CATTTCCACGATTTCCCAGA-3'
m*Mx1*	Forward	5′-ATGGATTCTGTGAATAATCTGTGCA3′
	Reverse	5′-CTATGTCTCCAAACTGGGAAGGG3′
m*Isg15*	Forward	5’-AGAAGCAGATTGCCCAGAAG-3’
	Reverse	5’-TGCGTCAGAAAGACCTCATAGA-3’
m*Ifit1*	Forward	5’-CCTAAACAGTTACTCCACCTTCG-3’
	Reverse	5’-TTGCTGACCTCCTCCATTCT-3’
