# Supplementary material for: ZC3HAV1 facilitates STING activation and enhances inflammation
Source: Commun Biol. 2024 Oct 30;7:1418. doi: 10.1038/s42003-024-07116-2 (PMC11526107; doi:10.1038/s42003-024-07116-2)
Supplement: Supplementary file 2 — Description of Additional Supplementary File [file 42003_2024_7116_MOESM2_ESM.pdf]

Description Of Additional Supplementary File

File name: Supplementary Data 1

Description: The source data behind the graphs in the paper
